# Inflammatory cytokine IL‐6 regulates ADAMTS14 expression through MAPK and PI3K signaling in colorectal cancer

**DOI:** 10.1002/ccs3.70092

**Published:** 2026-06-17

**Authors:** Nelin Hacioglu, Rümeysa Nur Vapur Ondul, Ghufran Haqi Ismael, Feray Kockar

**Affiliations:** ^1^ Faculty of Science and Literature Department of Molecular Biology and Genetics Balikesir University Balikesir Turkey

**Keywords:** ADAMTS14, extracellular matrix (ECM), interleukin‐6 (IL‐6), SW480, transcriptional regulation

## Abstract

Extracellular matrix (ECM) remodeling is a critical component of colorectal cancer (CRC) progression and tumor microenvironment organization. Members of the A Disintegrin and Metalloproteinase with Thrombospondin Motifs (ADAMTS) metalloproteinase family are known regulators of ECM structure; however, the transcriptional regulation of ADAMTS14 and its potential role in inflammation‐associated ECM remodeling remain poorly understood. In this study, we investigated whether inflammatory signaling regulates ADAMTS14 expression and explored its association with ECM organization in CRC. Interleukin‐6 (IL‐6) stimulation significantly increased ADAMTS14 expression at both mRNA and protein levels in CRC cells. Promoter deletion analyses identified a critical IL‐6‐responsive region between −145 and −43 bp upstream of the transcription start site, suggesting transcriptional responsiveness of ADAMTS14 to inflammatory signaling. Inhibition experiments demonstrated that Extracellular Signal‐Regulated Kinase, c‐Jun N‐terminal Kinase, Phosphatidylinositol 3‐Kinase, and Nuclear Factor Kappa B pathways were associated with IL‐6‐induced ADAMTS14 expression. Transcriptomic analyses of The Cancer Genome Atlas CRC datasets revealed that ADAMTS14 expression is elevated in tumors and is associated with inflammatory signaling, stromal activation, fibroblast‐related gene expression, and ECM organization pathways. Functional enrichment analyses indicated that ADAMTS14‐correlated genes are primarily involved in ECM organization, collagen fibril organization, and connective tissue development. Together, these findings identify ADAMTS14 as an inflammation‐responsive ECM‐associated metalloproteinase and suggest that IL‐6 signaling may be associated with ECM‐related transcriptional programs through regulation of ADAMTS14 expression. Our findings further suggest that ADAMTS14 expression may be associated with inflammatory and stromal‐related transcriptional programs in CRC.

## Graphical abstract

Interleukin‐6 induces ADAMTS14 expression in colorectal cancer cells. ADAMTS14 is transcriptionally regulated at the promoter level. Mitogen‐Activated Protein Kinase, Phosphatidylinositol 3‐Kinase, and Nuclear Factor Kappa B pathways mediate interleukin‐6‐induced ADAMTS14 expression. ADAMTS14 promotes extracellular matrix remodeling and stromal activation.

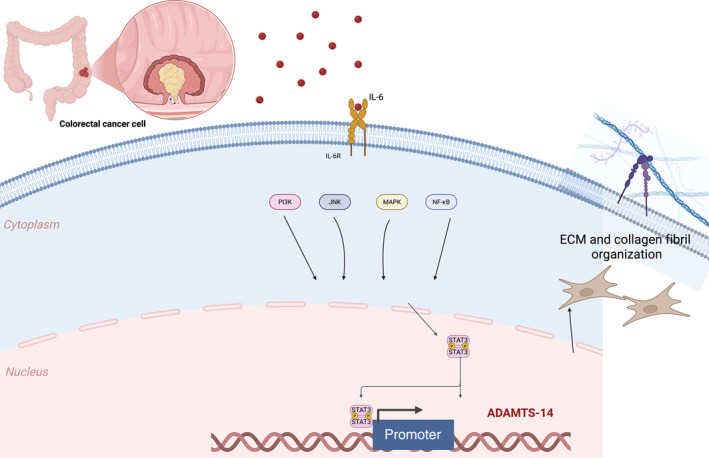

## 1 INTRODUCTION

Colorectal cancer (CRC) is one of the most common malignancies worldwide and represents a major global health burden due to its high incidence and mortality rates. It accounts for approximately 10% of all cancer cases and remains among the leading causes of cancer‐related deaths globally.^1^, ^2^ Epidemiological studies indicate that the global burden of CRC is expected to increase substantially in the coming decades largely due to demographic changes, population aging, and lifestyle‐associated risk factors such as obesity, sedentary behavior, and high consumption of processed and red meat. In addition to environmental factors, genetic and epigenetic alterations play a critical role in CRC development and progression.^3^ Although genetic and epigenetic alterations are central to CRC initiation, increasing evidence highlights the critical role of the tumor microenvironment in disease progression.

A key component of the tumor microenvironment is the extracellular matrix (ECM), a dynamic and highly regulated network that not only provides structural support but also actively modulates tumor cell behavior. Remodeling of the ECM is a fundamental process in cancer progression, influencing cell proliferation, migration, invasion, and metastatic dissemination.^4^, ^5^ In CRC, alterations in ECM composition and organization contribute to tumor‐stroma interactions and create a permissive environment for tumor progression. Therefore, understanding the molecular mechanisms that regulate ECM remodeling is essential for understanding tumor progression.

Among the major regulators of ECM remodeling are the ADAMTS (A Disintegrin and Metalloproteinase with Thrombospondin Motifs) family of secreted zinc‐dependent metalloproteinases. These enzymes play essential roles in ECM turnover, collagen processing, and tissue remodeling and are involved in diverse biological processes including angiogenesis, inflammation, and tumor progression.^6^, ^7^, ^8^ Dysregulation of ADAMTS family members has been increasingly associated with cancer development and progression, highlighting their importance in tumor‐associated ECM dynamics.

ADAMTS14 belongs to the procollagen N‐proteinase subgroup together with ADAMTS2 and ADAMTS3, which are critical for collagen maturation and ECM assembly.^9^, ^10^ Although these enzymes share structural and functional similarities, emerging evidence suggests that ADAMTS14 may exhibit distinct biological roles depending on tissue context. Altered expression of ADAMTS14 has been reported in multiple tumor types, including oral squamous cell carcinoma, clear cell renal cell carcinoma, and CRC.^11^, ^12^, ^13^, ^14^ Notably, both upregulation and downregulation of ADAMTS14 have been linked to tumor progression, indicating context‐dependent functions. However, the upstream regulatory mechanisms controlling ADAMTS14 expression in cancer, particularly within the tumor microenvironment and inflammatory context, remain poorly understood.

Inflammatory signaling is a major driver of tumor microenvironment remodeling, and cytokines play a central role in coordinating these processes. Among them, interleukin‐6 (IL‐6) is a pleiotropic cytokine that regulates immune responses, cell survival, and proliferation and has been widely implicated in cancer‐associated inflammation.^15^ IL‐6 signals through the Interleukin‐6 receptor (IL‐6R) and gp130 complex, leading to activation of multiple intracellular pathways, including Janus Kinase (JAK)/STAT, Mitogen‐Activated Protein Kinase (MAPK), and Phosphatidylinositol 3‐Kinase (PI3K)/Protein Kinase B (AKT).^16^ These signaling cascades not only promote tumor cell growth and survival but also contribute to remodeling of the tumor microenvironment, including ECM dynamics.^17^ Therefore, inflammatory signaling may represent an important upstream regulator of ECM‐associated proteases.

Despite the well‐established roles of IL‐6 signaling and ECM remodeling in cancer, the potential link between inflammatory cytokine signaling and ADAMTS14‐mediated matrix regulation has not been clearly defined. In particular, whether IL‐6 signaling can influence ADAMTS14 expression and thereby link inflammatory signaling to ECM remodeling remains unknown. In this study, we investigated whether IL‐6 regulates ADAMTS14 expression in CRC cells. Using the SW480 cell line, we examined the effects of IL‐6 on ADAMTS14 expression at transcriptional, mRNA, and protein levels. Promoter deletion analyses were performed to identify regulatory regions responsible for cytokine responsiveness, and the involvement of key intracellular signaling pathways was evaluated using pharmacological inhibitors in combination with quantitative real‐time Polymerase Chain Reaction (qRT‐PCR) and Western blot analyses. In addition, transcriptomic analyses were performed to evaluate the association between ADAMTS14 expression and inflammatory signaling, stromal activation, and ECM organization in CRC datasets. Through these approaches, this study aims to investigate the potential association between IL‐6‐responsive ADAMTS14 expression and ECM‐related transcriptional programs in CRC.

## 2 MATERIAL AND METHOD

### 2.1 Cell culture, cytokine treatment and inhibitor experiments

The human colorectal adenocarcinoma cell line SW480 (ATCC® CCL‐228™, RRID:CVCL_0546) was used in this study. This cell line was originally derived from a primary colon adenocarcinoma of a 50‐year‐old male patient (species: *Homo sapiens*; tissue of origin: colon adenocarcinoma, epithelial morphology). SW480 cells were obtained from the American Type Culture Collection (ATCC, Manassas, VA, USA) and maintained in High Glucose Dulbecco’s Modified Eagle Medium supplemented with 10% fetal bovine serum at 37°C in a humidified incubator with 5% CO_2_ (Tokay & Kockar, 2016c).^18^ To investigate the effect of inflammatory signaling on ADAMTS14 expression, cells were treated with recombinant human IL‐6. For pathway analysis, cells were pretreated with specific signaling inhibitors targeting Extracellular Signal‐Regulated Kinase (ERK) (PD98059), p38 MAPK (PD169316), c‐Jun N‐terminal Kinase (JNK) (SP600125), NF‐κB inhibitor, and PI3K (Wortmannin) prior to IL‐6 stimulation.^19^

Cell line authentication was confirmed based on supplier documentation, cell morphology, and growth characteristics consistent with SW480 cells. The SW480 cell line is not listed among commonly misidentified or contaminated cell lines in the International Cell Line Authentication Committee database.

### 2.2 Cloning ADAMTS14 promoter constructs and transient transfection

The genomic sequence of the human ADAMTS14 gene was retrieved from the National Center for Biotechnology Information database based on the reference genome (NC_000010.11) and transcript annotation (NM_139058.4) to design promoter constructs. To investigate transcriptional regulation, a series of promoter fragments with different upstream lengths relative to the transcription start site were generated. Specifically, three fragments spanning −381/+297, −145/+297, and −43/+297 were designed to identify putative regulatory regions responsible for cytokine responsiveness. Promoter regions were amplified by PCR using SW480 cell as a template. Primer sets were designed to incorporate appropriate restriction enzyme recognition sites at the 5′ ends to facilitate directional cloning (Table 1). Following amplification, PCR products were purified and initially cloned into a suitable intermediate vector. Subsequently, inserts were subcloned into the pMetLuc luciferase reporter vector, enabling functional analysis of promoter activity. All constructs were verified by sequencing to confirm insert integrity and correct orientation. These promoter‐reporter constructs were then used in transient transfection experiments to assess IL‐6‐dependent transcriptional activation of the ADAMTS14 gene.^20^, ^21^, ^22^

**TABLE 1:** ADAMTS14 promoter cloning primers.

ADAMTS14 promoter cloning primers	Product length
F1: 5′ GAGGGAGTCTCCCTCTA ′3	678 bp (−381/+297)
F2: 5′ GATCCCCGGCCGCAGGC ′3	442 bp (−145/+297)
F3: 5′ TGCCCACCCCCGGCCCGT ′3	340 bp (−43/+297)
R: 5′ AGCCATGTGGCCGCGCTGG ′3	

### 2.3 Calcium phosphate mediated transient transfection

ADAMTS14 promoter activity was analyzed using deletion constructs containing −381/+297, −145/+297 and −43/+297 regions relative to the transcription start site. SW480 cells were transfected with the respective promoter constructs using the calcium phosphate precipitation method. Briefly, plasmid DNA was mixed with CaCl_2_ and added dropwise to 2× HEPES‐buffered saline to form DNA‐calcium phosphate complexes, which were then applied to the cells. After 16 h incubation, the medium was replaced with fresh culture medium and cells were reseeded into 96‐well plates (3 × 10^4^ cells/well). For pathway inhibition experiments, cells were pretreated with for 1 h specific inhibitors PD98059 (20 μM), SP600125 (20 μM), PD169316 (20 μM), or Wortmannin (2 μM) prior to IL‐6 stimulation (20 ng/mL). After 6 h of treatment, culture supernatants were collected and stored at −20°C for luciferase and Secreted Alkaline Phosphatase (SEAP) activity measurements (Tokay & Kockar, 2016c).^19^

### 2.4 Luciferase and SEAP activity assay

Luciferase reporter activity was measured from culture supernatants using a luminometer according to the manufacturer's protocol. Briefly, culture medium was mixed with luciferase substrate and luminescence was recorded. SEAP activity was measured in parallel and used for normalization of transfection efficiency. Relative luciferase activity was calculated as the ratio of luciferase to SEAP signals. Data were analyzed using GraphPad Prism software.

### 2.5 RNA isolation and quantitative real‐time PCR

Total RNA was isolated from SW480 CRC cells using the GeneJET RNA Purification Kit (Thermo Fisher Scientific). RNA concentration and purity were determined by measuring absorbance at 260 and 280 nm. Complementary DNA was synthesized from 1 μg total RNA using reverse transcriptase and oligo (dT) primers. QRT‐PCR was performed using SYBR Green Master Mix with gene‐specific primers on a real‐time PCR system (Table 2). Gene expression levels were normalized to the housekeeping gene β2‐microglobulin and calculated using the 2^−ΔΔ*Ct*^ method.^23^, ^24^, ^25^, ^26^ All reactions were performed in triplicate.

**TABLE 2:** qPCR primers.

Primers
ADAMTS14 F: 5′ CCCTCTACTTCAATGTCACT 3′
ADAMTS14 R: 5′ GGTACACCA CATGTGTCCT 3′
B2M F: 5′ TTTCTGGCCTGGAGGCTATC 3′
B2M R: 5′ CATGTCTCGATCCCACTTAACT 3′

Abbreviation: B2M, Beta‐2‐microglobulin.

### 2.6 Protein extraction and western blot analysis

SW480 CRC cells were seeded in 6‐well plates at a density of 2.5 × 10^5^ cells per well and allowed to attach. Cells were then incubated in serum‐reduced medium containing 1% Bovine Serum Albumin (BSA) for 6 h prior to stimulation. For cytokine treatment experiments, cells were stimulated with recombinant human IL‐6 (20 ng/mL) for the indicated time points (1, 3, 6, 24, and 48 h). For signaling pathway inhibition experiments, cells were treated with IL‐6 in the presence of specific inhibitors targeting ERK (PD98059), p38 MAPK (PD169316), JNK (SP600125), PI3K (Wortmannin), or Nuclear Factor Kappa B (NF‐κB) signaling pathways. Following treatments, cells were washed with ice‐cold Phosphate‐Buffered Saline (PBS) and lysed using Radioimmunoprecipitation Assay Buffer lysis buffer supplemented with protease inhibitors. Cell lysates were briefly sonicated and centrifuged at 13,500 rpm for 15 min at 4°C to remove debris. The supernatants containing total proteins were collected and protein concentrations were determined using the Bradford assay. Equal amounts of protein were mixed with Laemmli sample buffer, denatured at 95°C for 5 min, and separated by Sodium Dodecyl Sulfate Polyacrylamide Gel Electrophoresis using 10% polyacrylamide gels. Proteins were subsequently transferred onto Polyvinylidene Difluoride membranes using a wet transfer system. Membranes were blocked in blocking buffer and incubated overnight at 4°C with primary antibodies against ADAMTS14 (PA5‐103578, Invitrogen) and β‐actin loading control (A3854, Sigma Aldrich). After washing with Tris‐Buffered Saline with Tween‐20, membranes were incubated with appropriate Horseradish Peroxidase‐conjugated secondary antibodies for 1 h at room temperature. Protein bands were detected using enhanced chemiluminescence reagents and visualized using a Fusion FX imaging system. Band intensities were quantified using ImageJ software, and ADAMTS14 protein levels were normalized to β‐actin.^27^ Quantitative analyses were performed using GraphPad Prism software.

### 2.7 Immunofluorescence analysis

Intracellular localization of ADAMTS14 protein in SW480 CRC cells was analyzed using immunofluorescence. Cells were plated in 24‐well plates with a sterile round coverslip at the base, at a density of 5 × 10^4^ cells per well, and incubated overnight at 37°C with 5% CO_2_ to ensure cell adhesion. The following day, cells were stimulated with IL‐6 (20 ng/mL) and incubated for the indicated time. At the end of incubation, cells were washed twice with PBS and fixed with 4% paraformaldehyde for 15 min. Following fixation, cells were washed with PBS and permeabilized with PBS containing 0.25% Triton X‐100 for 10 min. To reduce nonspecific binding, cells were blocked with PBS containing 1% BSA for 90 min. Next, the primary antibody against ADAMTS14 was diluted 1:300 in PBS containing 1% BSA and added to the cells, which were then incubated overnight at 4°C. The following day, after washing the cells with PBS, they were incubated with Alexa Fluor 488 conjugated secondary antibody (1:400 in 1% BSA) in the dark for 45 min. The cells were then washed with PBS and incubated with 4′,6‐Diamidino‐2‐Phenylindole (DAPI) (1 μg/mL) for 10 min for nuclear staining. The slide preparations were mounted using antifade solution and imaged using a fluorescence microscope.^22^ Fluorescence signals for ADAMTS14 were recorded, and the images were analyzed using ImageJ software.

### 2.8 Bioinformatic analysis

Transcriptomic data and corresponding clinical information for colorectal adenocarcinoma were obtained from The Cancer Genome Atlas (TCGA‐COAD) cohort through the Genomic Data Commons data portal.^28^ Gene expression values were log_2_‐transformed prior to downstream analyses. Differential expression of ADAMTS14 between tumor and normal tissues was evaluated using the Wilcoxon rank‐sum test.^29^ For survival analysis, patients were stratified into high and low ADAMTS14 expression groups based on the median expression value, and overall survival differences were evaluated using Kaplan–Meier analysis.^30^ Correlation analyses were performed to assess the association between ADAMTS14 expression and IL6/Signal Transducer and Activator of Transcription 3 (STAT3) signaling components as well as stromal activation markers including Platelet‐Derived Growth Factor Receptor Beta, Fibroblast Activation Protein (FAP), ACTA2, and Cluster of Differentiation 163 (CD163). Spearman correlation analysis was used to evaluate associations between gene expression variables unless otherwise indicated.^31^ Gene Set Enrichment Analysis (GSEA) was performed to identify biological pathways associated with ADAMTS14 expression using curated gene sets related to IL6–JAK–STAT3 signaling and inflammatory response pathways.^32^ Additionally, gene ontology (GO) enrichment analysis was conducted to identify biological processes associated with ADAMTS14‐related genes. All statistical analyses and data visualization were performed in R software (version 4.5.1) using the packages ggplot2, survival, survminer, clusterProfiler, and enrichplot together with GraphPad Prism.^33^, ^34^, ^35^, ^36^

### 2.9 Statistical analysis

Statistical analyses were performed using GraphPad Prism (GraphPad Software) and R software. Data are presented as mean ± standard deviation from at least three independent biological experiments unless otherwise stated. Correlation analyses were performed using Spearman correlation analysis. Exact *p* values are reported where available, and *p* < 0.05 was considered statistically significant. One‐way Analysis of Variance followed by Tukey's multiple comparison test was used for comparisons involving more than two groups.

## 3 RESULTS

### 3.1 Increased ADAMTS14 expression in colon cancer and its relationship with the IL6/JAK/STAT3 signaling pathway

**FIGURE 1:**
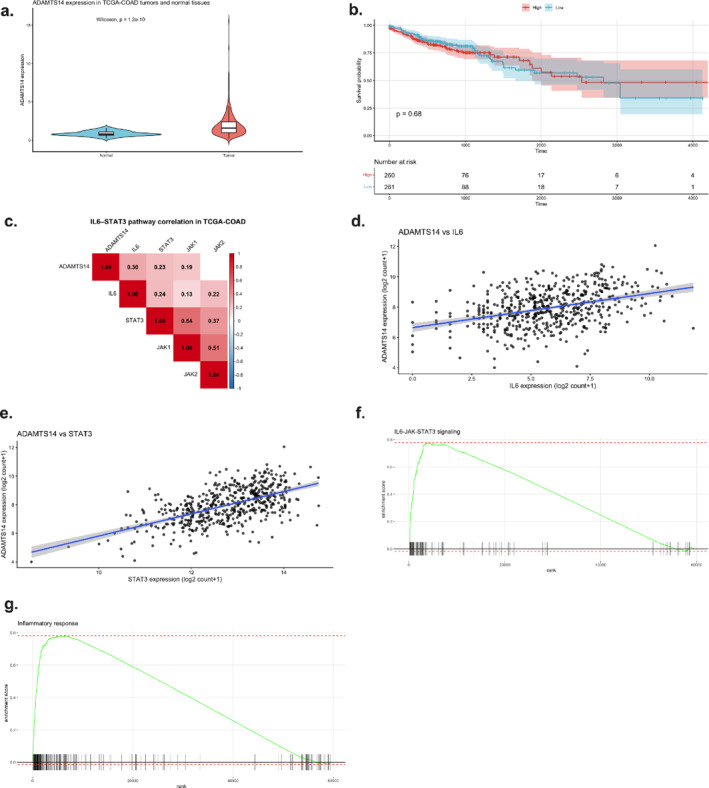
Analysis of the relationship between ADAMTS14 expression and the clinical and IL6/JAK/STAT3 signaling pathways in the TCGA‐COAD dataset. (A) Comparison of ADAMTS14 gene expression levels in normal colon tissues and tumor tissues in the TCGA‐COAD dataset. Statistical analysis was performed using the Wilcoxon test. (B) Overall survival analysis according to high and low ADAMTS14 expression groups was performed using the Kaplan–Meier method. (C) Correlation analysis between ADAMTS14, IL6, STAT3, JAK1, and JAK2 genes was performed using the Pearson correlation test and shown as a correlation matrix. (D) Correlation analysis between ADAMTS14 and IL6 gene expressions in the TCGA‐COAD dataset. (E) Correlation analysis between ADAMTS14 and STAT3 gene expressions in the TCGA‐COAD dataset. (F) GSEA analysis of the IL6‐JAK‐STAT3 signaling pathway between high and low expression groups of ADAMTS14. (G) GSEA analysis of the inflammatory response gene set between high and low expression groups of ADAMTS14. GSEA, Gene Set Enrichment Analysis; JAK, Janus Kinase.

To determine the clinical and molecular significance of the ADAMTS14 gene in colon cancer, the TCGA‐COAD dataset was analyzed. First, ADAMTS14 gene expression was compared between tumor and normal tissues. The analysis results showed that ADAMTS14 expression was significantly increased in tumor tissues compared to normal tissues (Wilcoxon, *p* = 1.2e‐10) (Figure 1A). This result indicates that the ADAMTS14 gene is upregulated in colon cancer. To evaluate the prognostic significance of ADAMTS14 gene expression, a Kaplan–Meier survival analysis was performed. No significant difference was observed in overall survival between the high and low ADAMTS14 expression groups (*p* = 0.68) (Figure 1B). This result suggests that ADAMTS14 expression alone may not be a strong prognostic marker. To evaluate the relationship between the IL‐6 signaling pathway and ADAMTS14, a correlation analysis was performed between ADAMTS14, IL6, STAT3, JAK1, and JAK2 genes. The correlation matrix results revealed that ADAMTS14 gene expression showed a positive correlation with IL6, STAT3, and JAK kinases (Figure 1C). The positive correlation between ADAMTS14 and IL6 and STAT3 was particularly noteworthy. To confirm these findings, the correlation between ADAMTS14 and IL6 and STAT3 gene expressions was analyzed separately. The results showed that ADAMTS14 gene expression showed a positive correlation with both IL6 and STAT3 expression (Figure 1D,E). These results indicate that the IL‐6/STAT3 signaling pathway is associated with ADAMTS14 expression. A GSEA analysis was performed to determine whether the IL‐6 signaling pathway is active in high‐expression groups of ADAMTS14. GSEA results showed a significant enrichment of the IL6‐JAK‐STAT3 signaling pathway in the high‐expression group of ADAMTS14 (Figure 1F). Similarly, the inflammatory response gene set was also found to be significantly enriched in the high‐expression group of ADAMTS14 (Figure 1G).

Taken together, these findings suggest that ADAMTS14 expression is increased in colon cancer and is associated with IL6/JAK/STAT3 signaling and inflammatory response pathways. Based on these transcriptomic findings, we hypothesized that IL‐6 signaling may directly regulate ADAMTS14 expression. To test this hypothesis, we performed in vitro experiments to investigate the effect of IL‐6 on ADAMTS14 expression and its transcriptional regulation in CRC cells.

### 3.2 Determining the effect of IL‐6 application on ADAMTS14 gene expression

**FIGURE 2:**
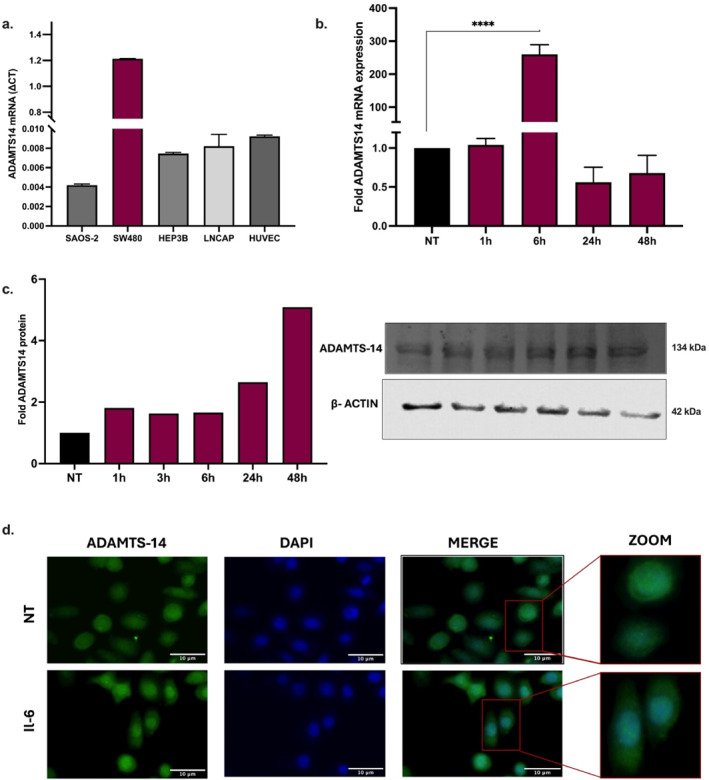
Determination of the effect of IL‐6 application on ADAMTS14 expression. (A) Basal mRNA expression levels of the ADAMTS14 gene in different cancer cell lines were analyzed by qRT‐PCR. (B) SW480 cells were treated with 20 ng/mL IL‐6, and ADAMTS14 mRNA expression levels were determined by qRT‐PCR after the specified time periods (1, 6, 24, and 48 h). (C) ADAMTS14 protein levels after IL‐6 application were analyzed by Western blot, and β‐actin was used as a loading control. Protein band densities were analyzed densitometrically, and the fold change is shown in the graph. Western blot analysis was performed from a single experimental replicate; therefore, no error bars or SD values are presented. (D) ADAMTS14 protein expression was examined by immunofluorescence staining. Nuclei were stained with DAPI. Scale bar: 10 μm. All experiments were performed in triplicate, and data are presented as mean ± SD. Western blot analysis was performed from a single experimental replicate. Statistical analysis was performed using ANOVA, with *p < 0.05, **p < 0.01, and ***p < 0.001 values. ANOVA, Analysis of Variance; DAPI, 4′,6‐Diamidino‐2‐Phenylindole; IL‐6, Interleukin‐6; qRT‐PCR, quantitative real‐time Polymerase Chain Reaction; SD, standard deviation.

To determine the basal expression levels of the ADAMTS14 gene in different cancer cell lines, ADAMTS14 mRNA expression was analyzed using qRT‐PCR in SAOS‐2, SW480, Hep3B, LNCaP, Human Umbilical Vein Endothelial Cells, and Pancreatic Cancer Cell Line (PANC‐1) cell lines (Figure 2A). The results showed that ADAMTS14 gene expression differed among cell lines and was expressed at a higher level in the SW480 CRC cell line compared to other cell lines (Supplementary Figure S1). Therefore, SW480 cells were selected as the model cell line for subsequent experiments. To determine the effect of IL‐6 on ADAMTS14 gene expression, 20 ng/mL IL‐6 was applied to SW480 cells, and ADAMTS14 mRNA expression levels were analyzed using qRT‐PCR after 1, 6, 24, and 48 h of incubation (Figure 2B). According to the results obtained, IL‐6 application was determined to increase ADAMTS14 mRNA expression in a time‐dependent manner. Specifically, ADAMTS14 mRNA expression increased statistically significantly at 6 h, reaching its highest expression level (*p* < 0.001). At longer incubation periods (24 and 48 h), ADAMTS14 expression decreased but remained higher compared to the control group. Western blot analysis was subsequently performed to evaluate ADAMTS14 protein levels following IL‐6 treatment (Figure 2C). Consistent with the mRNA expression profile, ADAMTS14 protein levels showed a gradual increase following IL‐6 stimulation, becoming more prominent at later time points, particularly at 24 and 48 h. Immunofluorescence staining was performed to evaluate changes in ADAMTS14 protein expression following IL‐6 treatment (Figure 2D). Compared to the control group, increased ADAMTS14 fluorescent signal intensity was observed in IL‐6‐treated cells. Nuclei were visualized using DAPI staining. These findings were consistent with the increased ADAMTS14 protein levels observed following IL‐6 stimulation and further support the association between IL‐6 treatment and elevated ADAMTS14 expression in SW480 cells.

### 3.3 IL‐6 activates ADAMTS14 promoter activity

**FIGURE 3:**
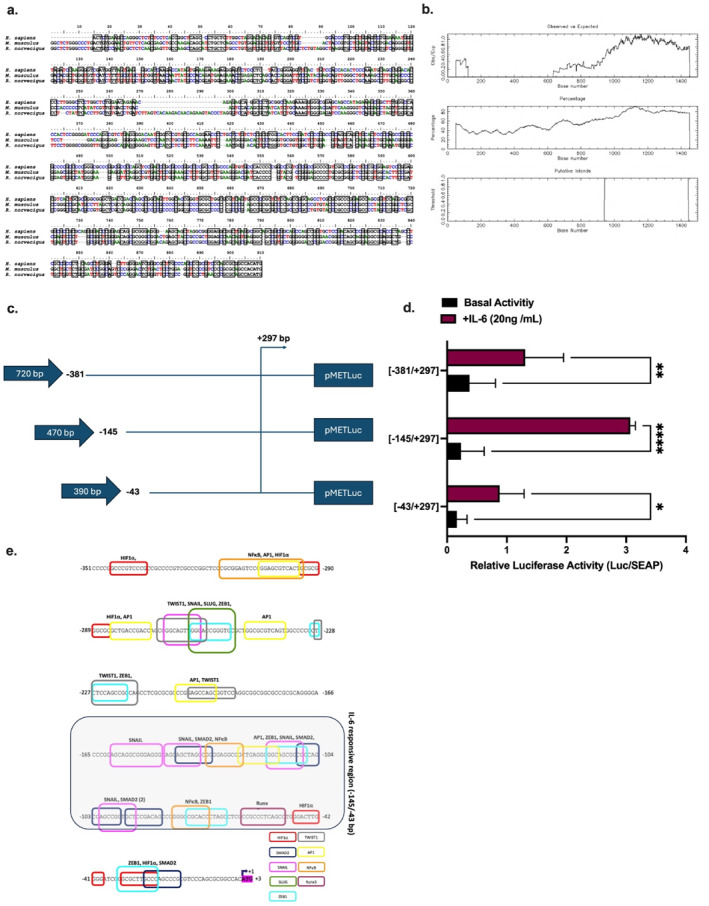
Sequence analysis of the ADAMTS14 promoter region, promoter deletion constructions, and the effect of IL‐6 on promoter activity. (A) Multiple sequence alignment analysis of human, mouse, and rat ADAMTS14 promoter regions. Conserved nucleotide regions are shown in shaded form. (B) Base composition and CpG island analyses of the ADAMTS14 promoter region are shown. (C) Schematic representation of 5′ deletion constructions (−381/+297, −145/+297, and −43/+297) generated from the ADAMTS14 promoter region and cloning of these regions into the pMetLuc report vector. (D) Determination of the effect of IL‐6 (20 ng/mL) administration on ADAMTS14 promoter activity in SW480 cells by luciferase report analysis. Luciferase activity was normalized by SEAP. Data are presented as mean ± SD. *p < 0.05, **p < 0.01, ***p < 0.001. (E) Schematic representation of predicted transcription factor binding motifs within the ADAMTS14 promoter region. The experimentally identified IL‐6‐responsive region (−145/−43 bp) is highlighted in gray. In silico promoter analysis identified multiple putative NF‐κB, AP‐1, SMAD2‐associated binding motifs clustered within this region, suggesting a transcriptionally active regulatory hotspot potentially involved in IL‐6‐responsive ADAMTS14 regulation. IL‐6, Interleukin‐6; NF‐κB, Nuclear Factor Kappa B; SEAP, Secreted Alkaline Phosphatase.

To investigate the transcriptional regulation of the ADAMTS14 gene, the ADAMTS14 promoter region was cloned and functionally characterized for the first time. The promoter region was analyzed in silico, and multiple sequence alignment analysis of human, mouse, and rat ADAMTS14 promoter sequences revealed highly conserved regions across species (Figure 3A). These conserved regions suggest the presence of potential regulatory elements that may have functional significance for the transcriptional regulation of the ADAMTS14 gene. Base composition and CpG island analyses of the promoter region showed the presence of GC‐rich regions around the transcription start region, and these regions may be important for promoter activity (Figure 3B). For functional promoter analyses, promoter regions of varying lengths of the ADAMTS14 gene were amplified by PCR, cloned into the pGEM‐T Easy vector, and then subcloned into the pMetLuc report vector to obtain luciferase report constructions. In this context, 5′ deletion promoter constructs containing the −381/+297, −145/+297, and −43/+297 regions were created (Figure 3C). The promoter activities of these constructs were measured using the luciferase reporting system both at baseline and after IL‐6 (20 ng/mL) administration. Luciferase analysis results showed that IL‐6 administration significantly increased promoter activity in the −381/+297 and especially the −145/+297 promoter constructs. In contrast, the increase in IL‐6‐dependent promoter activity was significantly reduced in the −43/+297 construct (Figure 3D). In silico promoter analysis further identified multiple putative NF‐κB‐, AP‐1‐, SMAD‐, and Hypoxia‐Inducible Factor 1 Alpha‐related binding motifs within the IL‐6‐responsive −145/‐43 bp promoter region, supporting the potential regulatory relevance of this segment in cytokine‐responsive ADAMTS14 expression (Figure 3E). These results indicate that the key regulatory region responding to IL‐6 is located between base pairs −145 and −43. When all these findings are considered together, it has been determined that the ADAMTS14 promoter region has been cloned and functionally characterized for the first time, and that the ADAMTS14 gene is transcriptionally regulated by IL‐6 with the key promoter region responsible for this regulation located in the range of −145/‐43.

### 3.4 MAPK and PI3K signaling pathways contribute to IL‐6‐mediated ADAMTS14 activation

**FIGURE 4:**
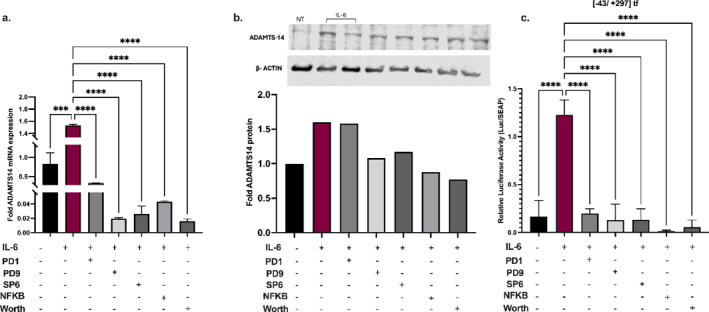
Identification of signaling pathways involved in IL‐6‐mediated ADAMTS14 regulation. (A) SW480 cells were treated with 20 ng/mL IL‐6 alone or in combination with different signaling pathway inhibitors (PD98059: ERK inhibitor, PD169316: p38 MAPK, SP600125: JNK inhibitor, Wortmannin: PI3K inhibitor). ADAMTS14 mRNA expression levels were analyzed by Quantitative real‐time PCR after treatment. (B) Under the same conditions, ADAMTS14 protein levels were analyzed by Western blot, and β‐actin was used as a loading control. Protein band densities were analyzed densitometrical, and fold change is shown in the graph. (C) Luciferase report gene analysis was performed using the ADAMTS14 promoter construction containing the −43/+297 promoter region, and the effect of inhibitor administration in combination with IL‐6 on promoter activity was determined. Luciferase activity was calculated as the Luc/SEAP ratio. All experiments were performed in triplicate, and data are presented as mean ± standard deviation. Western blot analysis was performed from a single experimental replicate. Statistical analysis was performed using ANOVA, with *p < 0.05, **p < 0.01, and ***p < 0.001. ANOVA, Analysis of Variance; ERK, Extracellular Signal‐Regulated Kinase; IL‐6, Interleukin‐6; MAPK, Mitogen‐Activated Protein Kinase; PI3K, Phosphatidylinositol 3‐Kinase; SEAP, Secreted Alkaline Phosphatase.

To determine which intracellular signaling pathways IL‐6 uses to regulate ADAMTS14 gene expression, SW480 cells were treated with IL‐6 alone or in combination with different signaling pathway inhibitors, and ADAMTS14 expression levels were analyzed at the mRNA, protein, and promoter activity levels (Figure 4). Examination of qRT‐PCR results showed that IL‐6 application significantly increased ADAMTS14 mRNA expression. However, when IL‐6 was applied in combination with ERK inhibitor (PD98059), p38 MAPK inhibitor (PD169316), JNK inhibitor (SP600125), and PI3K inhibitor (Wortmannin), ADAMTS14 mRNA expression levels were significantly decreased (Figure 4A). These results indicate that ERK, JNK, PI3K, and MAPK signaling pathways play a role in IL‐6‐mediated ADAMTS14 gene expression. Western blot analysis results were also consistent with qRT‐PCR results (Figure 4B). IL‐6 administration increased ADAMTS14 protein levels, whereas inhibitor administration caused a significant decrease in ADAMTS14 protein levels. Densitometric analysis results showed that inhibitor administration suppressed ADAMTS14 protein expression. This indicates that IL‐6 regulates ADAMTS14 expression both transcriptionally and protein‐wise through these signaling pathways. To determine the effect of IL‐6 on ADAMTS14 promoter activity and the role of inhibitors on this activity, luciferase report gene analysis was performed (Figure 4C). IL‐6 administration was found to significantly increase ADAMTS14 promoter activity, whereas the presence of ERK, JNK, PI3K, and MAPK inhibitors significantly decreased promoter activity. These results demonstrate that IL‐6 regulates the ADAMTS14 gene at the transcriptional level, and this regulation occurs via the ERK, JNK, PI3K, and MAPK signaling pathways. Considering all these findings together, it is concluded that IL‐6 regulates ADAMTS14 gene expression through multiple intracellular signaling pathways, and these signaling pathways play a significant role in the transcriptional activation of the ADAMTS14 gene.

### 3.5 Relationship of ADAMTS14 expression with stromal activation, fibroblast markers, and inflammation

**FIGURE 5:**
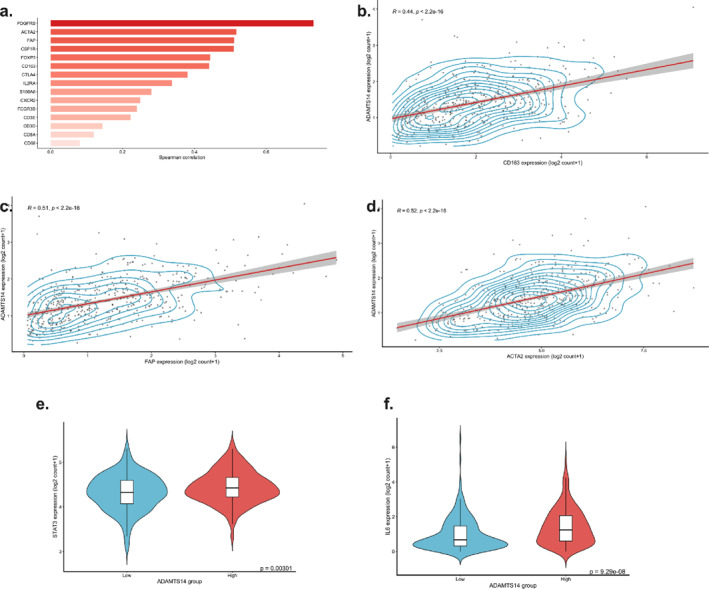
Analysis of the association of ADAMTS14 expression with tumor microenvironment, stromal activation, and inflammation. (A) Spearman correlation analysis between ADAMTS14 expression and stromal and fibroblast‐related genes in the TCGA‐COAD dataset. (B–D) Spearman correlation analyses between ADAMTS14 expression and the expression of stromal/fibroblast markers CD163, FAP, and ACTA2 genes. (E) Comparison of STAT3 gene expression levels in low and high ADAMTS14 expression groups. (F) Comparison of IL6 gene expression levels in low and high ADAMTS14 expression groups. Statistical analyses were performed using the Spearman correlation test and the Wilcoxon test. CD163, Cluster of Differentiation 163; FAP, Fibroblast Activation Protein.

To evaluate the relationship of the ADAMTS14 gene with the tumor microenvironment, a Spearman correlation analysis was performed between ADAMTS14 expression and genes associated with stromal activation and fibroblasts in the TCGA‐COAD dataset. The correlation analysis results showed that ADAMTS14 expression showed a positive Spearman correlation particularly with genes associated with stromal activation and fibroblasts (Figure 5A). To further investigate these findings, correlation analyses were conducted between ADAMTS14 expression and the stromal and tumor‐associated fibroblast markers CD163, FAP, and ACTA2 genes. The results showed a significant and positive correlation between ADAMTS14 expression and CD163, FAP, and ACTA2 expression (Figure 5B–D). These results suggest that high ADAMTS14 expression may be associated with stromal activation and the presence of tumor‐associated fibroblasts. To evaluate the relationship between ADAMTS14 expression and inflammatory signaling pathways, STAT3 and IL6 gene expressions were compared in low and high ADAMTS14 expression groups. Analysis results showed significantly higher STAT3 and IL6 expression levels in the high ADAMTS14 expression group (Figure 5E,F). These findings indicate that ADAMTS14 expression is associated with inflammatory response and IL‐6/STAT3 signaling pathway activity.

Considering all these findings together, it is thought that high ADAMTS14 expression is associated with stromal activation, fibroblast markers, and inflammatory signaling pathway activity, and that ADAMTS14 expression was associated with stromal activation and fibroblast‐related transcriptional signatures.

### 3.6 Relationship of ADAMTS14 expression to ECM organization, EMT, and inflammation

**FIGURE 6:**
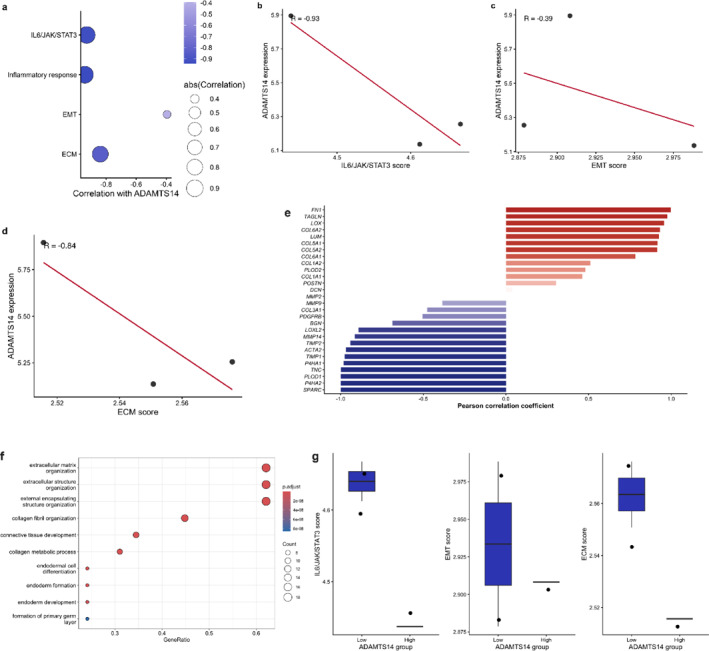
Analysis of the relationship between ADAMTS14 expression and ECM organization, EMT, and inflammation. (A) Spearman correlation analysis between ADAMTS14 expression and IL6/JAK/STAT3 signaling pathway, inflammatory response, EMT, and ECM scores. (B–D) Spearman correlation analyses between ADAMTS14 expression and IL6/JAK/STAT3 score, EMT score, and ECM score. (E) Ranking of genes showing positive and negative correlations with ADAMTS14 expression according to Spearman correlation coefficients. (F) Results of gene ontology enrichment analysis for genes correlated with ADAMTS14. (G) Comparison of IL6/JAK/STAT3 score, EMT score, and ECM score in low and high ADAMTS14 expression groups. ECM, extracellular matrix; EMT, Epithelial‐to‐Mesenchymal Transition; JAK, Janus Kinase.

To evaluate the relationship between ADAMTS14 gene expression and biological processes, Spearman correlation analyses were conducted between ADAMTS14 expression and IL6/JAK/STAT3 signaling pathway activity, inflammatory response, Epithelial‐to‐Mesenchymal Transition (EMT) score, and ECM score. The analysis results revealed a positive correlation between ADAMTS14 expression and the IL6/JAK/STAT3 signaling pathway and inflammatory response (Figure 6A). A strong positive Spearman correlation was identified between ADAMTS14 expression and the IL6/JAK/STAT3 score (Figure 6B). Conversely, a correlation was observed between ADAMTS14 expression and the EMT and ECM scores (Figure 6C,D). These findings indicate that ADAMTS14 expression is associated with inflammatory signaling activity, while showing distinct relationships with EMT‐ and ECM‐related transcriptional signatures.

When genes correlated with ADAMTS14 were examined, it was determined that most genes showing a positive correlation with ADAMTS14 were related to ECM organization and stromal structure. Conversely, genes showing a negative correlation were related to epithelial phenotype and cell adhesion (Figure 6E). The results of GO enrichment analysis performed on these genes showed that genes associated with ADAMTS14 were particularly enriched in biological processes such as ECM organization, collagen fibril organization, and connective tissue development (Figure 6F). When comparing the low and high expression groups of ADAMTS14, it was determined that the high expression group had higher IL6/JAK/STAT3 signaling pathway activity, whereas EMT and ECM scores differed between the groups (Figure 6G). Additional ssGSEA analyses further demonstrated enrichment of inflammatory response, TNFα/NF‐κB signaling, TGF‐β signaling, and EMT‐associated transcriptional programs in ADAMTS14‐high tumors (Supplementary Figure S2). Spearman correlation analyses additionally demonstrated positive associations between ADAMTS14 expression and inflammatory/stromal‐related pathway activity scores with the strongest correlation observed for EMT‐associated transcriptional programs (Supplementary Figure S2). When all these findings are considered together, it is thought that ADAMTS14 expression may be associated with broader inflammatory and stromal‐related transcriptional programs in CRC.

## 4 DISCUSSION

Inflammatory signaling plays a central role in CRC development and progression. Among inflammatory mediators, interleukin‐6 (IL‐6) is a well‐characterized cytokine that regulates tumor cell behavior through activation of multiple intracellular signaling pathways, including JAK/STAT, MAPK, and PI3K/AKT cascades.^15^, ^37^ These pathways influence gene expression programs involved in cell proliferation, survival, invasion, and tumor microenvironment remodeling. In this context, identifying genes regulated by IL‐6 signaling is important for understanding how inflammatory signaling contributes to CRC progression.

In the present study, IL‐6 stimulation resulted in a time‐dependent increase in ADAMTS14 expression in SW480 CRC cells. ADAMTS14 mRNA expression peaked at early time points following IL‐6 stimulation, whereas protein accumulation became more apparent at later time points. This temporal difference may reflect the delay between transcriptional activation and protein accumulation, a commonly observed feature of cytokine‐responsive gene regulation.^38^ Immunofluorescence analyses further supported these observations by demonstrating increased intracellular ADAMTS14 signal following IL‐6 stimulation.

To further investigate the transcriptional regulation of ADAMTS14, promoter deletion analyses were performed using a series of ADAMTS14 promoter constructs. These experiments revealed that IL‐6 stimulation enhanced ADAMTS14 promoter activity and that sequences located within the −145 to −43 region upstream of the transcription start site contribute to this response. In silico transcription factor binding site analyses identified potential STAT3, NF‐κB, and AP‐1‐associated motifs within this region, suggesting that it may contain putative IL‐6‐responsive regulatory elements. Although specific transcription factor binding was not experimentally validated in the present study, these findings support the presence of cytokine‐responsive regulatory sequences within the ADAMTS14 promoter region.^37^

Our results further indicate that multiple intracellular signaling pathways may participate in the regulation of ADAMTS14 expression following IL‐6 stimulation. Pharmacological inhibition experiments demonstrated that MAPK signaling pathways, particularly ERK and p38 MAPK, as well as PI3K signaling, were associated with IL‐6‐induced ADAMTS14 expression. These observations are consistent with previous studies highlighting the role of MAPK and PI3K pathways in mediating IL‐6‐dependent gene expression responses in cancer cells.^39^ Inhibition of JNK and NF‐κB related signaling also influenced ADAMTS14 expression to a lesser extent, suggesting that IL‐6‐associated regulation of ADAMTS14 may involve multiple signaling inputs, which is characteristic of cytokine signaling networks in cancer.^17^

To explore the potential clinical relevance of these findings, bioinformatic analyses were performed using CRC datasets from TCGA. These analyses revealed differential expression of ADAMTS14 in colorectal tumors and identified correlations between ADAMTS14 expression and components of the IL‐6/STAT3 signaling axis. In addition, gene set enrichment analyses indicated enrichment of inflammatory signaling pathways in tumors with elevated ADAMTS14 expression. Together, these observations suggest that ADAMTS14 expression may be associated with inflammatory signaling‐related transcriptional programs in CRC.

Further functional analyses revealed that genes co‐expressed with ADAMTS14 were primarily associated with ECM organization and stromal‐related transcriptional signatures. Notably, several genes correlated with ADAMTS14 expression, including CDH11, FAP, and ACTA2, are commonly used markers associated with tumor‐associated fibroblasts and stromal‐related processes.^40^ These findings are consistent with previous reports describing associations between ADAMTS family metalloproteinases and ECM dynamics.^6^, ^7^ Consistent with this observation, functional enrichment analyses indicated that ADAMTS14‐associated genes were significantly enriched in biological processes related to ECM organization, collagen fibril organization, and connective tissue development.

Interestingly, pathway activity analyses further demonstrated that ADAMTS14 expression was associated with inflammatory signaling activity, particularly IL‐6/JAK/STAT3 signaling, as well as ECM organization and epithelial‐mesenchymal transition‐related gene signatures. These findings suggest that ADAMTS14 expression may be linked to inflammatory signaling and ECM‐related transcriptional programs in CRC.

Collectively, the present study identifies ADAMTS14 as an IL‐6‐responsive gene in CRC cells and provides evidence that its expression is associated with IL‐6‐responsive promoter regions and multiple intracellular signaling pathways. In addition, transcriptomic analyses indicate that ADAMTS14 expression is associated with inflammatory signaling activity, stromal‐related transcriptional signatures, and ECM organization. To our knowledge, there are currently no studies describing the regulation of ADAMTS14 by cytokine signaling in CRC. In this context, our findings provide the first experimental evidence linking inflammatory cytokine signaling to ADAMTS14 expression and suggest that ADAMTS14 expression may be associated with inflammation‐related tumor microenvironment remodeling in CRC. Further studies will be required to clarify the functional consequences of ADAMTS14 regulation in colorectal tumor progression.

Although ADAMTS14 expression was associated with inflammatory and ECM‐related transcriptional programs, the present study does not directly establish a functional role for ADAMTS14 in ECM remodeling. Furthermore, the experiments were primarily conducted in the SW480 CRC model, and additional pathway analyses, ChIP‐qPCR validation, and multi cell line protein level validations were not performed.

## 5 CONCLUSION

This study demonstrates that ADAMTS14 is an inflammation‐responsive gene associated with IL‐6 signaling in CRC cells. IL‐6 stimulation increased ADAMTS14 expression at both mRNA and protein levels and enhanced ADAMTS14 promoter activity, indicating transcriptional responsiveness. Promoter deletion analysis identified the −145 to −43 bp region as a critical IL‐6‐responsive regulatory region. Inhibition experiments further showed that IL‐6‐induced ADAMTS14 expression was associated with multiple intracellular signaling pathways, including ERK, JNK, PI3K, and NF‐κB‐related signaling pathways. Bioinformatic analyses using TCGA CRC datasets revealed associations between ADAMTS14 expression and IL‐6/JAK/STAT3 signaling, inflammatory response pathways, stromal‐related transcriptional signatures, and ECM organization. In addition, ADAMTS14 expression was positively correlated with fibroblast‐related genes and ECM‐associated processes, suggesting a potential association with tumor microenvironment‐related transcriptional programs. Collectively, these findings support a potential association between IL‐6‐responsive ADAMTS14 expression and ECM‐related transcriptional signatures in CRC. This study provides new insight into the relationship between inflammatory signaling and ECM‐related transcriptional programs and suggests that ADAMTS14 expression may be associated with inflammation‐related tumor microenvironment remodeling in CRC.

## AUTHOR CONTRIBUTIONS

**Nelin Hacioglu:** Conceptualization; bioinformatic analysis; data curation; writing—original draft; grant acquisition. **Rümeysa Nur Vapur Ondul**: Investigation; methodology; data collection. **Ghufran Haqi Ismael**: Investigation; methodology; data collection. **Feray Kockar** Supervision; writing—review and editing. All authors commented on previous versions of the manuscript and approved the final version.

## CONFLICT OF INTEREST STATEMENT

The authors declare no conflicts of interest.

## ETHICS STATEMENT

This article does not contain research in which animals or humans were used, so ethical approval was not required for this study.

## Supporting information

Supporting Information S1

## Data Availability

The data that support the findings of this study are available from the corresponding author upon reasonable request.
