# Supplementary material for: Inflammatory cytokine IL‐6 regulates ADAMTS14 expression through MAPK and PI3K signaling in colorectal cancer
Source: J Cell Commun Signal. 2026 Jun 17;20(2):e70092. doi: 10.1002/ccs3.70092 (PMC13276287; doi:10.1002/ccs3.70092)
Supplement: Supplementary file 1 — Supporting Information S1 [file CCS3-20-e70092-s001.docx]

**
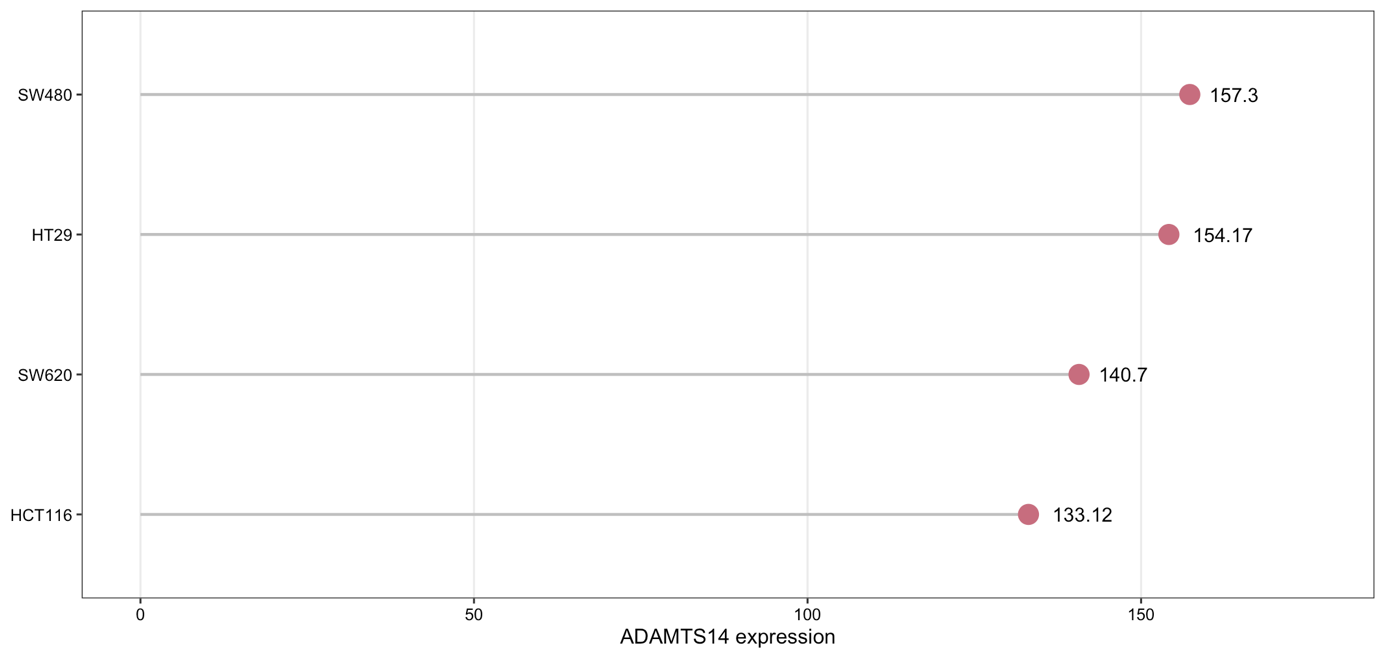
Supplementary Figure S1.** ADAMTS14 expression across colorectal cancer cell lines in the GSE59857 dataset.


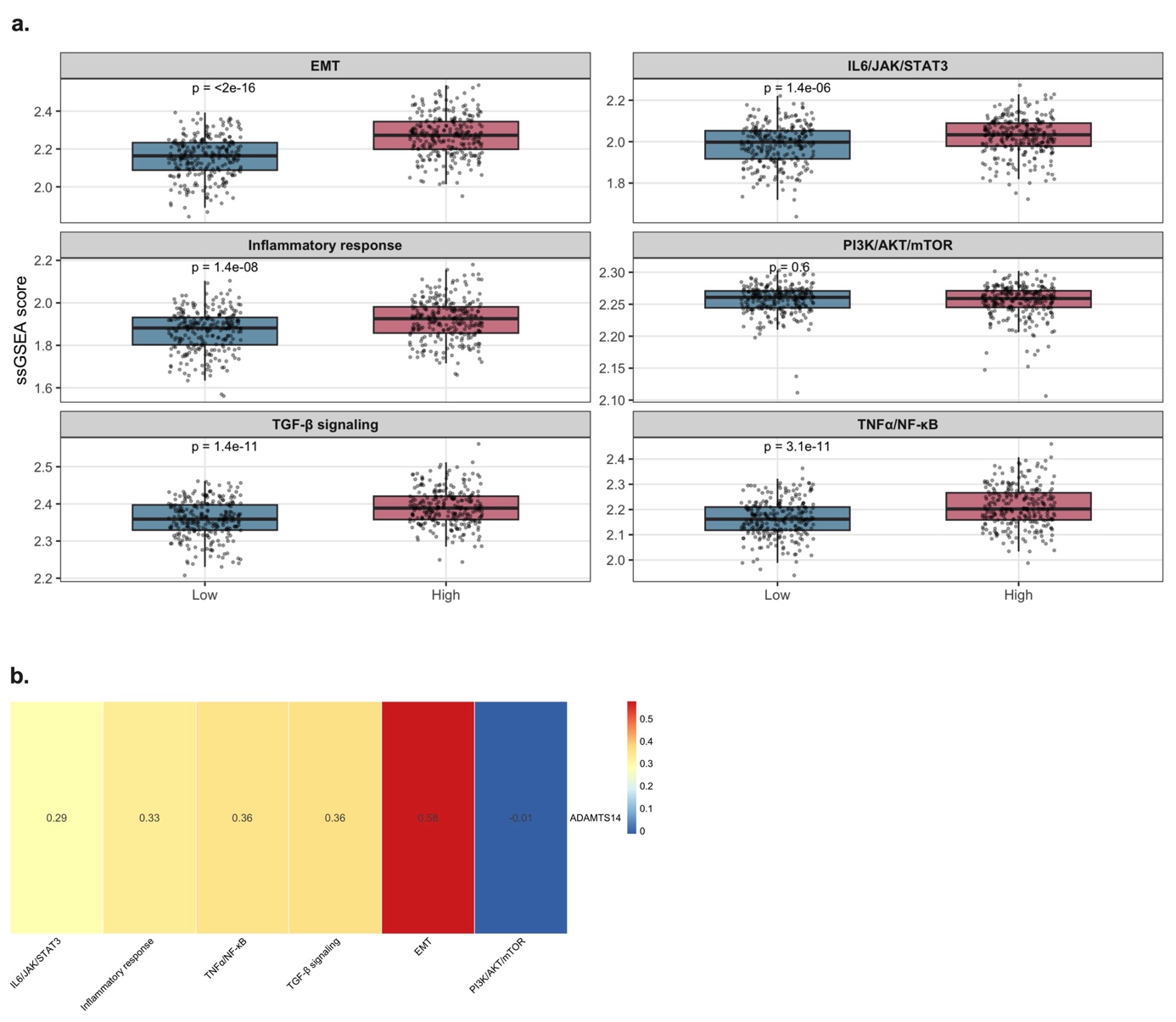


**Supplementary Figure S2.** (a) Comparative ssGSEA pathway activity analysis between ADAMTS14-low and ADAMTS14-high colorectal cancer samples demonstrated significantly elevated EMT, IL6/JAK/STAT3 signaling, inflammatory response, TGF-β signaling, and TNFα/NF-κB pathway activities in the ADAMTS14-high group, whereas PI3K/AKT/mTOR signaling showed no significant difference.

(b) Correlation heatmap illustrating the association between ADAMTS14 expression and pathway activity scores. ADAMTS14 expression showed the strongest positive correlation with EMT-related transcriptional programs, followed by TGF-β signaling, TNFα/NF-κB signaling, inflammatory response, and IL6/JAK/STAT3 activity, while no meaningful correlation was observed with PI3K/AKT/mTOR signaling.
